# Industrially-Scalable Microencapsulation of Plant Beneficial Bacteria in Dry Cross-Linked Alginate Matrix

**DOI:** 10.1089/ind.2017.0032

**Published:** 2018-06-01

**Authors:** Scott A. Strobel, Kimberly Allen, Christopher Roberts, Desmond Jimenez, Herbert B. Scher, Tina Jeoh

**Affiliations:** ^1^ Department of Biological and Agricultural Engineering, University of California-Davis, Davis, CA; ^2^ NewLeaf Symbiotics, St. Louis, MO

**Keywords:** Industrial microbiology, living organisms, chemistry of natural biopolymers, agriculture

## Abstract

*Microencapsulation of plant-beneficial bacteria, such as pink pigmented facultative methylotrophs (PPFM), may greatly extend the shelf life of these Gram-negative microorganisms and facilitate their application to crops for sustainable agriculture. A species of PPFM designated* Methylobacterium radiotolerans *was microencapsulated in cross-linked alginate microcapsules (CLAMs) prepared by an innovative and industrially scalable process that achieves polymer cross-linking during spray-drying. PPFM survived the spray-drying microencapsulation process with no significant loss in viable population, and the initial population of PPFM in CLAMs exceeded 10^10^ CFU/g powder. The PPFM population in CLAMs gradually declined by 4 to 5 log CFU/g over one year of storage. The extent of alginate cross-linking, modulated by adjusting the calcium phosphate content in the spray-dryer feed, did not influence cell viability after spray-drying, viability over storage, or dry particle size. However, particle size measurements and light microscopy of aqueous CLAMs suggest that enhanced crosslinking may limit the release of encapsulated bacteria. This work demonstrates an industrially scalable method for producing alginate-based inoculants that may be suitable for on-seed or foliar spray applications.*

## Introduction

As knowledge of plant-microbe interactions expands, commercial interests aspire to harness these interactions to advance sustainable agriculture. While rhizobacteria such as *Azospirillum* and *Pseudomonas* have been applied to crops for decades to facilitate nitrogen-fixation and to enhance protection against plant pathogens,^1^ other rhizobacteria such as pink pigmented facultative methylotrophs (PPFMs) have only recently drawn attention for their beneficial interactions with plants and their potential application in modern agriculture.^2^

PPFMs are Gram-negative α-proteobacteria of the *Methylobacterium* genus that are known to colonize nodules and other plant tissue by using their ability to utilize single carbon substrates such as methanol, formaldehyde, and formate as a competitive advantage.^3^ Most of the >50 known PPFM species have only been identified over the last decade.^2^ While the benefits conferred to plants are specific to the plant and PPFM species, these benefits include participating in nitrogen fixation and nodule formation,^4^ enhancing seed germination and seedling growth,^5^ promoting plant growth,^6,7^ and providing protection against phytopathogens.^8,9^ As such, there is considerable interest in applying PPFMs to agricultural crops as natural biocontrol and growth-promoting agents. As an example, application of *Methylobacterium radiotolerans* to jatropha, a potential biodiesel crop, dramatically increased seed yield while also improving biomass production.^10^

However, applying plant-growth promoting bacteria to crops is not a trivial task. Currently, inoculation with plant growth promoting bacteria is most commonly practiced in the cultivation of legumes, which are inoculated with rhizobacteria either by directly applying a liquid formulation to soil or seeds or by applying solid-phase, peat-anchored bacteria to seed coats or pellets.^11^ Both solid and liquid conventional inoculant formulations suffer from relatively short shelf life and high transportation costs.^1^ The encapsulation of plant growth promoting bacteria in polymers such as alginate, which protect and gradually release the inoculant in soil, has been proposed as an alternative over the last 30 years.^12,13^ Other advantages of dried alginate beads as inoculants include the natural abundance of the seaweed-derived material, its biodegradability in soil, and its nontoxicity; in addition, the dried beads are easily stored and transported.^12,14,15^ While alginate macrobeads (diameter >1 mm) have been demonstrated to gradually release plant-growth-promoting bacteria in soil and improve plant growth,^12,16–20^ microscale alginate beads offer advantages, including adhesion to seed surfaces and more rapid release of bacterial cargo.^14,21^ However, production of alginate microbeads is low-throughput.^21^

Alginate inoculants are typically prepared by extruding or spraying a sodium alginate solution containing the microorganism into a calcium chloride solution. The calcium ions induce gelation through their electrostatic interaction with the negatively-charged carboxylic acid groups to form “egg-box” structures between the guluronate-rich regions of adjacent alginate molecules.^22^ A commercially viable procedure to scale up the production of dry alginate-encapsulated microorganisms has yet to be demonstrated. Spray-drying is a well-established and highly scalable microencapsulation technology that has been used in the food industry to encapsulate microbes in dry powder form.^23,24^ A few studies have explored spray-drying as a method to stabilize plant-growth promoting bacteria in a variety of materials, including alginate, but cross-linking of the alginate was not achieved.^25–27^ Spray-drying bacteria (such as probiotics) in cross-linked alginate requires a calcium gelation step before or after spray-drying.^28–31^ While spray-drying enables the production of large quantities of material, it is often considered an unsuitable method for cell microencapsulation due to the potential for high microbial mortality during simultaneous heating and dehydration.^1^

Recently, we utilized spray-drying to produce powdered cross-linked alginate microparticles and microcapsules.^32–34^ In this method, particle formation, drying, and cross-linking of the alginate occur in a single step. The alginate solution fed into the spray-dryer contains an insoluble calcium salt, an acid, and a volatile base such as ammonium hydroxide. After the nozzle atomizes the fluid into droplets, the base vaporizes in the hot gas current, acidifying the droplet fluid. At the lower pH, the calcium salt solubilizes and cross-links the alginate. This technology has previously been demonstrated for encapsulating enzymes^32^ and emulsified lipids.^33^

In this study, we investigated the extent to which Gram-negative bacteria, specifically PPFMs, can be stabilized in spray-dried cross-linked alginate microcapsules (CLAMs). The viability of PPFMs was closely tracked throughout the microencapsulation process, and the survival of PPFMs in CLAMs was monitored over one year of storage. The impact of matrix cross-linking on survival was explored by encapsulating PPFMs in CLAMs with varying calcium phosphate content. Additionally, the morphology, size, and distribution of viable cells within the PPFM-CLAMs were evaluated.

## Materials and Methods

### Materials

Low viscosity sodium alginate (A1112), Schiff's fuchsin sulfite reagent, sodium metabisulfite, periodic acid, glutamic acid, and phosphate buffered saline (PBS) were purchased from Sigma-Aldrich (St. Louis, MO). Dicalcium phosphate dihydrate, sodium citrate dihydrate, succinic acid, ammonium hydroxide, SYTO9 nucleic acid stain, propidium iodide, and isopropanol were purchased from Fisher (Fair Lawn, NJ). Bacto soytone was purchased from BD Difco BBL (Houston, TX). MilliQ water with a minimum resistivity of 18 MΩ-cm (Millipore, Billerica, MA) was used for all experiments.

The pink-pigmented methylobacteria used in this study were provided by NewLeaf Symbiotics Inc. (St Louis, MO). The strain is deposited at the Northern Regional Research Labs as NRRL Accession #B50930. Based on MALDI-TOF and 16S rRNA sequence (Accugenix^®^ Charles River, Wilmington MA), the isolate was designated as *Methylobacterium radiotolerans*. Whole genome sequence information (Ilumina Hi-Seq, MOgene Dx, St Louis, MO and Pacific Biosciences, Menlo Park), coupled with ANI score analysis suggests that this strain is closely related to the type strain designated as *M. radiotolerans*.

### Methods

#### Growth of microorganisms

Methylotrophic bacteria were grown using amended ammonia mineral salts media^35^ containing 15 g/L of glutamic acid and 10 g/L of Bacto soytone. Media were adjusted to pH 6.8 using 1 M NaOH. Frozen cell concentrates were used to inoculate 500 mL baffled Erlenmeyer flasks containing 300 mL of sterile media. Shake flask cultures were grown using a rotating shaker incubator at 200 rpm and 30°C for 38 hours. Whole broth was sampled aseptically in a biological hood for colony forming unit (CFU) enumeration. Log phase cultures were harvested by centrifugation at 5,000 rpm in a fixed angle rotor Avanti centrifuge (ThemoFisher, Waltham, MA) for 5 min at 4°C. After centrifugation, the cell pellets were re-suspended in 300 mL of an alginate solution described below.

#### Preparation of spray-dried powders

Dry cross-linked alginate microcapsules (CLAMs) were prepared by spray-drying a well-mixed suspension of 2.0% (w/w) sodium alginate, 1.0% (w/w) succinic acid (adjusted to pH 5.6 with ammonium hydroxide), and insoluble calcium phosphate dibasic dihydrate. To achieve different extents of calcium alginate cross-linking, the concentration of calcium phosphate dibasic dihydrate in the spray-dryer inlet suspension was either 0.5% or 0.1% (w/w). The microorganism pellet was dispersed in this inlet suspension, which was subsequently pumped into a Buchi B290 laboratory spray-dryer (New Castle, DE) to produce dry, bacteria-loaded microcapsules. All formulations were prepared under identical operating conditions: inlet air temperature was set to 130°C, aspirator airflow rate was set to maximum (35 m^3^/h), peristaltic pump was set to 45% of maximum, and nozzle air flow was set to 50 mm on the Q-flow indicator. Under these conditions, the outlet temperatures ranged from 49–53°C. Triplicate lots of each powder (0.1% and 0.5% CaHPO_4_) were prepared and analyzed in triplicate. Spray dried powders were stored at room temperature in clear glass vials, with exposure to ambient light.

#### Enumeration of microorganisms

To enumerate the PPFM in CLAMs, 10 mg of powder was dissolved in 1.0 mL of phosphate buffered saline solution (137 mM NaCl, 2.7 mM KCl, 10 mM Na_2_HPO_4_, 2 mM KH_2_PO_4_, pH 7.4). Serial dilutions were performed, and 1.0 mL of each dilution was plated in triplicate onto solid minimal media composed of Glutamine/Bacto Soytone amended with 1.5% Bacto agar. To determine stability of encapsulated bacteria over storage, cell enumeration was performed periodically over 12 months. CFU/g values calculated for each powder batch replicate were transformed to log CFU/g and then averaged.

To determine the PPFM population in the shake flask, supernatant, and spray-dryer inlet suspension, 1.0 mL of fluid was serially diluted and plated in triplicate. After 7 d of incubation at 30°C, colonies were enumerated on plates containing between 30 and 300 colonies.

#### Moisture content analysis

Moisture content of spray-dried powders and total solids of the spray-drying inlet suspension were both determined gravimetrically after a 3-d drying period at 60–65°C.

#### Quantification of soluble alginate in aqueous CLAM suspensions

An assay to measure soluble alginate was adapted from Houghton et al.^36^ to assess alginate cross-linking based on the fraction of alginate that dissolves when CLAMs are suspended in water. To compare alginate release in water to total alginate for each batch of CLAMs, 0.1000 ± 0.0005 g of CLAM powder was weighed into 10 mL of water and also into 10 mL of 100 mM sodium citrate, in 15 mL conical tubes. After rotating for 2 h at 20 rpm at room temperature, a 1,000 μL sample was removed from each tube and centrifuged at 17,000 × g. The supernatants were sampled and diluted 50X in water. Alginate standards with concentrations of 0.30, 0.25, 0.20, 0.15, 0.10, 0.05, and 0.025 mg/mL were prepared from a 0.50 mg/mL stock solution. Each sample and standard were added in 200 μL volumes to a 96 well microtiter plate (n = 4). To each well was added 30 μL of 0.067% periodic acid in 4.67% acetic acid. The plate was incubated for 1 h at 37°C along with a diluted Schiff's fuchsin sulfite reagent (two parts per each part water) prepared with 16.7 mg sodium metabisulfite per mL of Schiff's fuchsin reagent. After the incubation, 30 μL of the diluted Schiff's fuchsin sulfite reagent was added to each well, and the plate was incubated for 1 h at room temperature for color development. The absorbance at 550 nm was measured using a plate reader (Biotek, model Synergy 4). The soluble-to-total alginate percentage for each sample was the ratio of the concentration of alginate released into water to the concentration of alginate fully dissolved in 100 mM sodium citrate.

#### Particle size analysis

The particle size distributions of non-hydrated microcapsules were determined using a Mastersizer 2000 particle analyzer with Hydro μP dispersion unit (Malvern Instruments, Worcestershire, UK). Spray dried powders were dispersed in propan-2-ol and added to the dispersion unit according to manufacturer guidelines. A refractive index of 1.51 and an absorption index of 0.1 were assumed for the powder, and a general purpose spherical model was selected. Measurements were made while the dispersion unit pump was set to 2,500 rpm. Prior to each sample measurement, samples were sonicated in the dispersion unit at 50% intensity for 10 minutes to break up aggregates. Each sample was measured in triplicate (each replicate returned ten measurements), and Malvern software was used to calculate an average distribution from the 30 distributions. The reported size parameters are the average ± standard deviation for the three replicates.

In addition, particle size distributions were determined as powders hydrated and released PPFM. For this analysis, powders were suspended in water and added to the dispersion unit, with water as the dispersing fluid. Measurements were performed every 2 min as the particles circulated through the dispersion unit (no sonication).

#### Scanning electron microscopy

Powder samples were mounted on carbon tape and imaged directly using a FEI/Philips XL30 SFEG SEM operated at 5 kV in SED mode. To minimize the effects of surface charging of the non-coated samples, images were processed using a 16-frame averaging filter.

#### Viability staining of encapsulated microorganisms

Approximately 5 mg of spray-dried powder was suspended in 500 μL water and vortexed. Based on the recommendations for the LIVE/DEAD BacLite bacterial viability kit (Molecular Probes), 10 μL of 1.5 mM propidium iodide and 0.5 μL of 5 mM SYTO 9 were added to the suspension. After approximately 20 min of incubation time, the PPFM-CLAM suspension was mounted on a glass slide and viewed using a fluorescence microscope (Olympus, Center Valley, PA). The SYTO 9 dye, which stains bacteria indiscriminately, was viewed using the FITC channel. Propidium iodide, which stains dead bacteria characterized by compromised membranes, was viewed using the TRITC channel.

#### Statistical analysis

Data were reported as mean ± standard deviation. Two-way ANOVA and multiple comparison tests were used to assess statistical differences between groups. Statistical analysis was performed using Prism 7 (GraphPad). P-values less than 0.05 were deemed to be significant.

## Results and Discussion

### Microencapsulating PPFM in CLAMs with Tunable Cross-Linking

Inoculation of crops with PPFMs, including the model strain *M. radiotolerans*, could potentially boost crop yields.^10^ Microencapsulation of these bacteria facilitates their application in sustainable agricultural systems, providing a means to preserve their viability during storage and control their release. However, minimizing the cost of the microencapsulation process remains a key challenge.^37^ This study demonstrates a novel and highly scalable process to microencapsulate Gram-negative rhizobacteria in a cross-linked alginate matrix, using *M. radiotolerans* as a model PPFM. While microencapsulation in dry cross-linked alginate has been proposed for a variety of plant-beneficial bacteria, including PPFMs,^20^ the method employed in this study streamlines the microencapsulation process using existing industrial equipment.

**Fig. 1.**
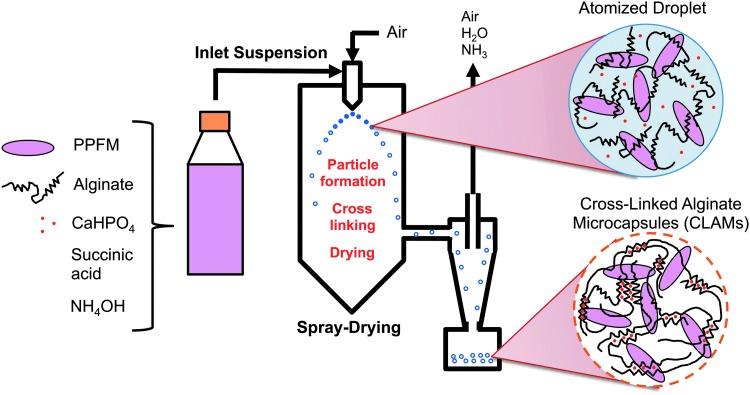
Schematic of the process for encapsulating microorganisms in cross-linked alginate microcapsules (CLAMs). During spray drying, the vaporization of ammonia reduces the droplet pH, making calcium ions available for cross-linking the alginate matrix.

In this process, a novel formulation facilitates the *in situ* cross-linking of alginate during spray-drying, effectively consolidating the series of time and energy intensive unit operations intrinsic to conventional alginate microbead preparation methods. To prepare cross-linked alginate microcapsules containing PPFMs (PPFM-CLAMs), a suspension of microorganisms, alginate, insoluble CaHPO_4_, and succinic acid (titrated with NH_4_OH to pH 5.6) was spray-dried (*Fig. 1*). During spray-drying, the suspension was atomized at the nozzle into minute droplets. As these droplets dried into microcapsules, the vaporization of the volatile base reduced the pH of the droplets, dissolving CaHPO_4_ and availing Ca-ions to cross-link the alginate.^32–34^ Thus, the bacteria in the feed stream exit the spray-dryer encapsulated in CLAMs in the form of a dry powder that is mostly insoluble in water. The scalability and industrial ubiquity of the spray-drying unit operation makes the CLAMs process particularly attractive for producing large quantities of microencapsulated plant-beneficial bacteria.

The CaHPO_4_ content of the spray-dryer inlet suspension was found to influence the extent of alginate cross-linking in the microcapsule matrix. Two sets of PPFM-CLAM powders were formulated with 0.1% insoluble CaHPO_4_ (0.1% Ca PPFM-CLAMs) and 0.5% CaHPO_4_ (0.5% Ca PPFM-CLAMs) in the spray-dryer inlet suspension, and the extent of cross-linking was measured as the percentage of total alginate that leached out from the PPFM-CLAMs when suspended in water for 2 h. The 0.5% Ca PPFM-CLAMs released 23.7 ± 2.0% of total alginate into solution, significantly less than the 52.7 ± 5.2% of total alginate released from 0.1% Ca PPFM-CLAMs, indicating that the 0.5% Ca PPFM-CLAMs contained a greater fraction of insoluble, cross-linked alginate, i.e., 0.5% Ca PPFM-CLAMs were more extensively cross-linked than the 0.1% Ca PPFM-CLAMs. The extent of cross-linking had no significant influence on the wet basis moisture content (6.60 ± 0.26% and 6.98 ± 0.67% for 0.1% Ca and 0.5% Ca PPFM-CLAMs, respectively) and the recovery yield of powder relative to dry solids in the feed (51.8 ± 2.2% and 54.3 ± 1.7% for 0.1% Ca and 0.5% Ca PPFM-CLAMs, respectively).

### Physical Characterization of PPFM-CLAMs

**Fig. 2.**
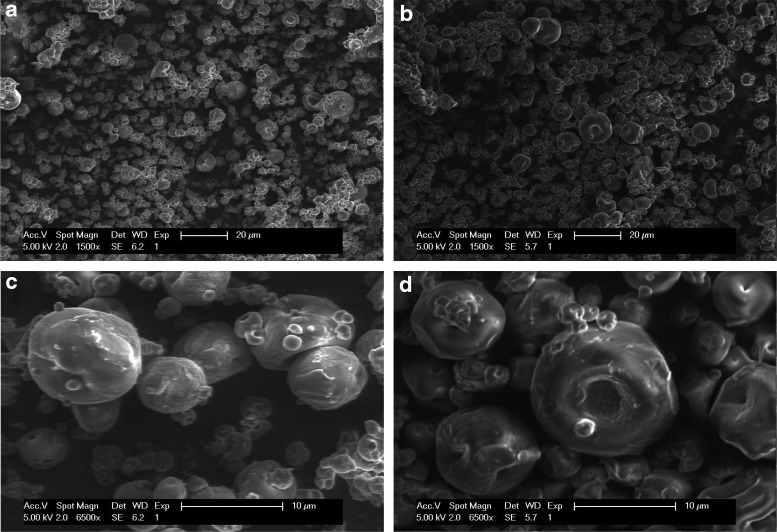
SEM images of PPFM-CLAMs. CLAMs were prepared with **(a, c)** 0.1% CaHPO_4_ and **(b, d)** 0.5% CaHPO_4_. Scale bars are **(a, b)** 20 μm and **(c, d)** 10 μm.

Scanning electron micrographs show that dry PPFM-CLAMs ranged in size from approximately 1–15 μm (*Fig. 2*). PPFM-CLAMs generally exhibited spherical morphology. Cross-linking level did not appear to influence particle morphology. Particle surfaces appeared dented, but no collapsed, cracked, or otherwise damaged particles were observed. While the surfaces of PPFM-CLAMs did not appear smooth, there was no evidence of non-encapsulated rod-shaped bacteria on particle surfaces or elsewhere.

**Fig. 3.**
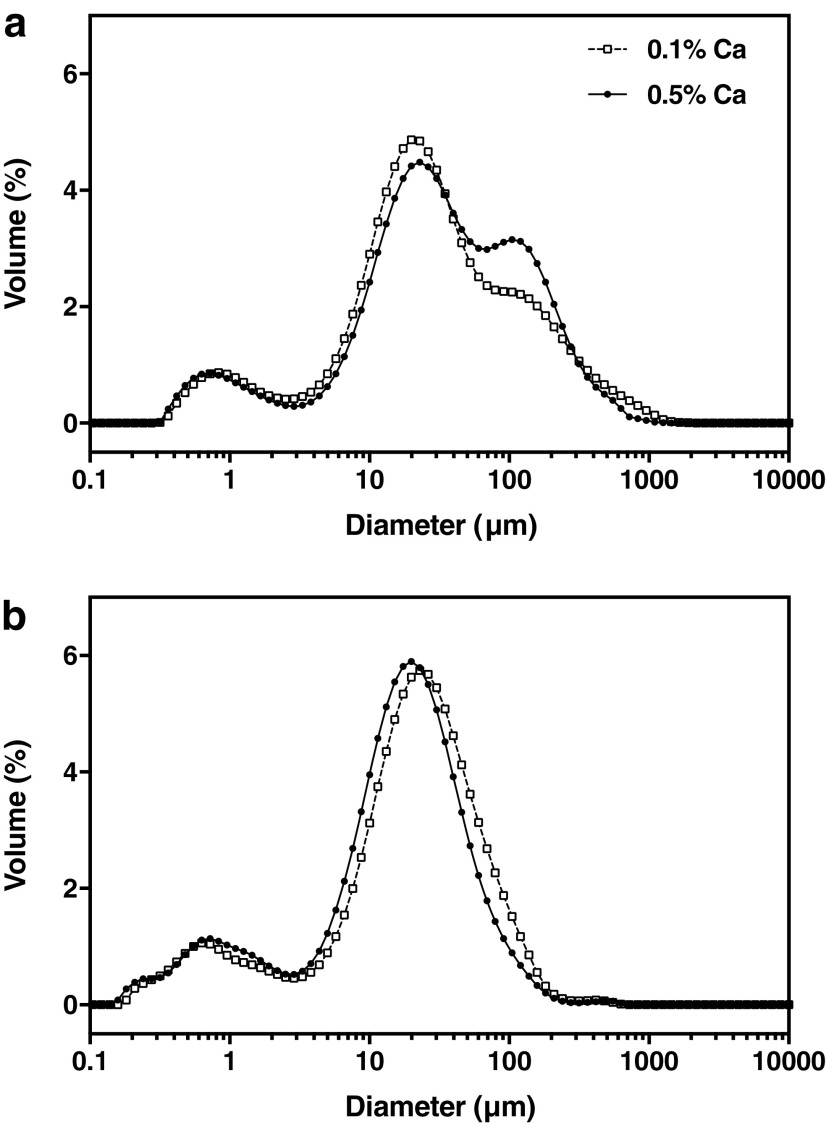
Particle size distributions of PPFM-CLAMs. Without sonication prior to measurement, size distributions of PPFM-CLAMs exhibited a shoulder in the 100 μm range **(a)**, which was not observed in size distributions measured after 10 minute sonication period **(b)**.

PPFM-CLAMs tended to form particle aggregates, as evidenced by the shoulders spanning from approximately 100–1,000 μm in the particle size distributions (*Fig. 3a*). Applying sonication to the dispersed PPFM-CLAMs narrowed the particle size distributions by eliminating the shoulder (*Fig. 3b*), yielding particle size measurements that generally agree with SEM observations (*Fig. 2*). The sonication step reduced the 90^th^ percentile diameter from 183 ± 35 μm to 66 ± 13 μm and from 171 ± 14 μm to 51 ± 8 μm for 0.1% and 0.5% Ca PPFM-CLAMs, respectively. CaHPO_4_ content had minimal effect on the particle size of PPFM-CLAMs. A fine particle size is desirable when developing dry alginate-based inoculants. For plant inoculation via foliar spray, achieving a fine particle size is an important consideration, as hydrated CLAMs would need to be small enough to pass through a nozzle. For on-seed applications, reducing alginate microbead size to the sub-millimeter range facilitates uniform seed coating and increases the release efficiency of encapsulated bacteria.^14,21^

### Release of Bacteria From PPFM-CLAMs

**Fig. 4.**
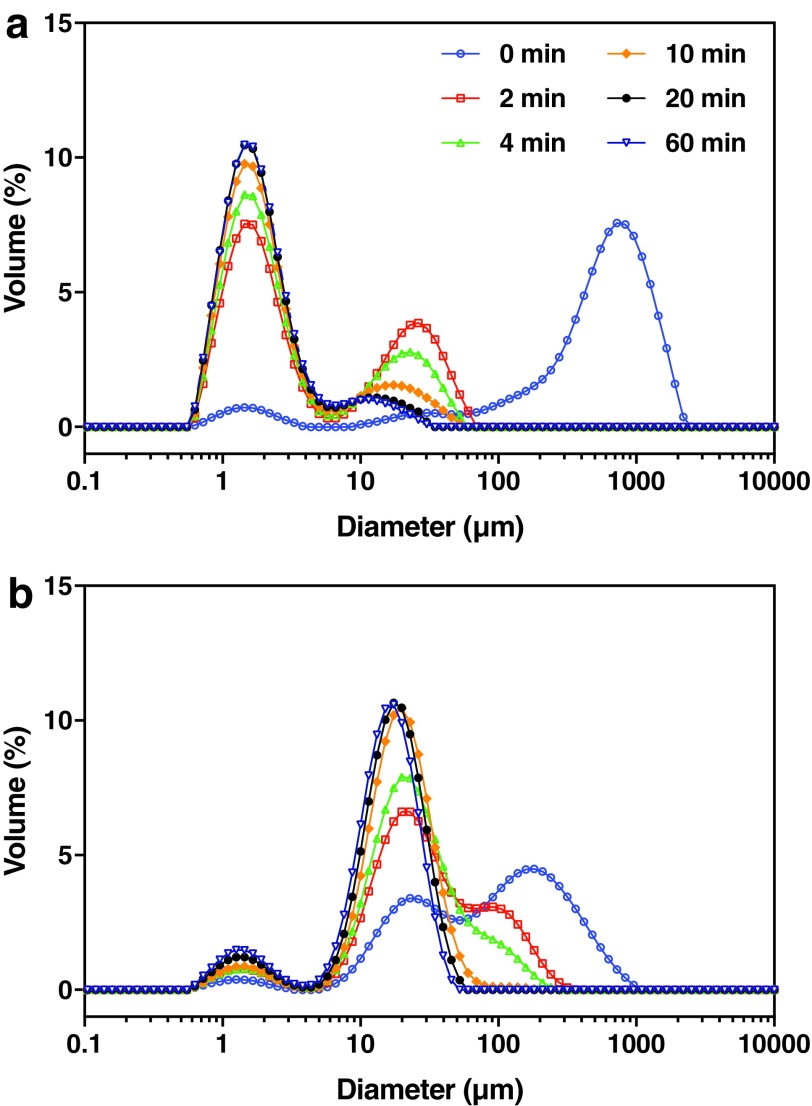
Change in particle size distributions of PPFM-CLAMs suspended in water. Over time, the size distributions for 0.1% Ca PPFM-CLAMs and 0.5% Ca PPFM-CLAMs both converged to bimodal distributions with peaks in the 1–2 μm and 10–20 μm range. **(a)** For 0.1% Ca PPFM-CLAMs, the 1–2 μm peak dominated the equilibrium size distribution. **(b)** The 10–20 μm peak dominated the equilibrium size distribution for 0.5% Ca PPFM-CLAMs.

The extent of alginate cross-linking did not affect particle size, but it appeared to govern the release of PPFMs from CLAMs. When suspended in water, the size distributions of 0.5% and 0.1% Ca PPFM-CLAMs each converged to bimodal distributions within approximately 20 min of continuous measurement (*Fig. 4*). The size distributions for PPFM-CLAMs featured two peaks centered at 10–20 μm and 1–2 μm, likely corresponding to the PPFM-CLAMs and PPFMs released into the fluid, respectively. For the moderately cross-linked 0.1% Ca PPFM-CLAMs after 20-min hydration, the 1–2 μm bacteria peak was of greater magnitude relative to the 10–20 μm peak corresponding to the microcapsules (*Fig. 4a*). The distribution's convergence to the bacteria peak suggested that the moderately cross-linked 0.1% Ca PPFM-CLAMs may rapidly break down in water and release a considerable portion of their cargo. Comparatively, 0.5% Ca PPFM-CLAMs appeared to release fewer bacteria, as the peak corresponding to the bacteria remained low in magnitude relative to the peak corresponding to the microcapsules (*Fig. 4b*). Thus, a more extensively cross-linked alginate matrix appeared to limit the immediate release of cargo, most likely because less of the alginate matrix solubilized into the surrounding solution (23.7 ± 2.0% of the alginate matrix compared to 52.7 ± 5.2% for 0.1% Ca PPFM-CLAMs).

**Fig. 5.**
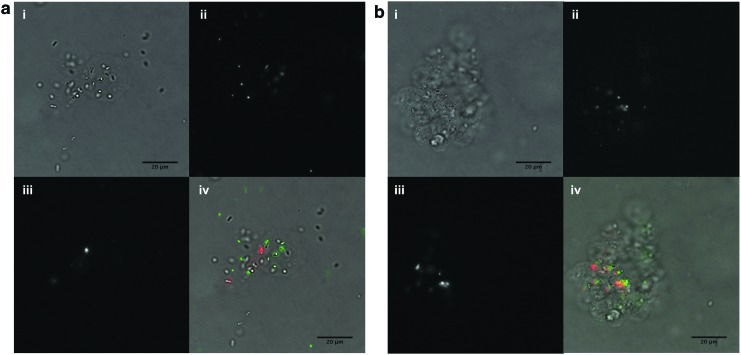
Light microscopy of PPFM-CLAMs. The 0.1% Ca PPFM-CLAMs **(a)** were less visible in the bright field channel (i) compared to 0.5% Ca PPFM-CLAMs **(b)**. Fluorescent viability staining shows cells indiscriminately in the FITC channel (ii) and cells with compromised membranes (presumed dead) in the TRITC channel (iii). The overlay of bright field and fluorescence channels is also shown (iv).

Fluorescence microscopy was used to visualize PPFMs within CLAMs, assess their viability, and verify release behavior (*Fig. 5*). Live cells appeared to outnumber dead cells. PPFMs were localized within CLAMs, but some cells did not appear to be visibly encapsulated; these cells were either released from CLAMs or were microencapsulated in thin coatings indistinguishable from the cell membranes. Fewer non-encapsulated bacteria were observed in the aqueous suspension of 0.5% Ca PPFM-CLAMs, supporting the observation that increased cross-linking reduced cargo release. Furthermore, PPFM-CLAMs prepared with 0.5% Ca were more easily observed in the bright field channel compared to 0.1% Ca CLAMs, suggesting that highly cross-linked CLAMs remained more physically intact.

### No Significant Loss of PPFM Viability During Spray-Drying

**Fig. 6.**
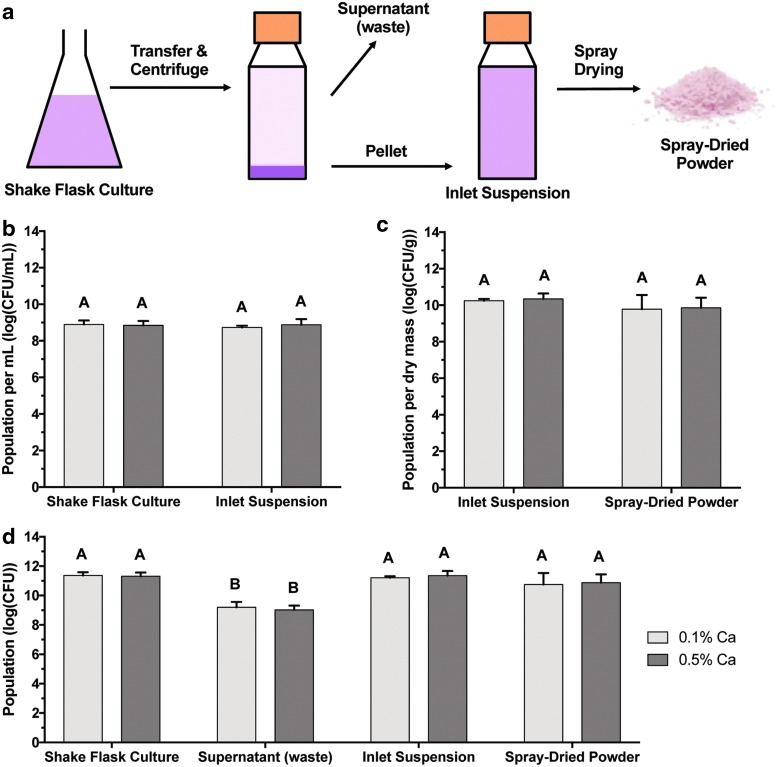
Schematic for PPFM-CLAMs preparation and PPFM enumeration. PPFM culture was grown in a shake flask and centrifuged. The pellet was transferred to a suspension consisting of 2% alginate, 1% succinic acid (pH 5.6), and either 0.1% CaHPO_4_ or 0.5% CaHPO_4_, which was subsequently spray-dried to form powdered microcapsules **(a)**. PPFM were enumerated (on a log(CFU/mL) basis) in the shake culture and after the pellet was resuspended in an equal volume of alginate suspension prior to spray-drying **(b)**. PPFM were enumerated (on a log(CFU/g) dry solids basis) before and after spray-drying **(c)**. To facilitate same-basis comparisons, the total population of PPFM, i.e., (CFU) = (CFU/mL) × (total volume in mL) or (CFU) = (CFU/g) × (total dry solids sprayed, in g), was determined at each stage of processing **(d)**. Error bars indicate standard deviations for triplicate powder lots. Within each panel, the same letter indicates no significant difference between means (α = 0.05).

The viability of PPFMs was tracked over the course of the microencapsulation process. The microbial population was enumerated in the shake flask culture, in the inlet suspension containing the cell pellet dispersed in the alginate solution, in the waste supernatant from the shake flask, and in the spray-dried powder (*Fig. 6a*). Shake flask cultures were consistently grown to approximately 10^9^ CFU/mL, and the microbial population per fluid volume was not significantly reduced when the cell pellet was resuspended in the same volume of alginate solution (*Fig. 6b*). The spray-dried 0.1% Ca and 0.5% Ca PPFM-CLAMs contained 1.2 × 10^10^ CFU/g and 1.1 × 10^10^ CFU/g, respectively, adjusted for moisture content (*Fig. 6c*). This constituted a 0.46 ± 0.73 and 0.48 ± 0.29 log CFU/g reduction relative to the population per dry mass in the spray-dryer inlet suspensions used to prepare the 0.1% and 0.5% Ca PPFM-CLAMs, respectively. Thus, the spray-drying step did not significantly reduce the viable PPFM population in either group. The total microbial population was calculated at each step of the microencapsulation process to provide a common basis for comparison (*Fig. 6d*). There was no significant difference in the total PPFM populations in the shake culture growth media, spray-dryer inlet suspension, and resultant powder; however, the population in the waste supernatant was two orders of magnitude lower than the population in the shake flask, indicating that cell losses during the separation step were negligible. The concentration of CaHPO_4_ in the inlet suspension did not significantly influence the survival of PPFMs at any stage of the microencapsulation process.

When developing inoculant formulations, the factors governing success include both the final bacterial population per dry mass and the fraction of surviving bacteria. The loading of viable PPFMs in the spray-dried powders (>10^10^ CFU/g) was comparable to loadings in dry alginate microbeads (prepared by the conventional external gelation method) that others have used for seed inoculation.^13,14^ For Gram-positive bacteria (e.g., probiotics such as *Bifidobacteria* and *Lactobacilli*) spray-dried with alginate, surviving populations exceeding 10^10^ CFU/g have been reported.^30,38,39^ The present study demonstrates similar bacterial populations surviving spray-drying, with the considerable benefit of avoiding costly additional processing steps associated with alginate cross-linking.

The spray-drying step did not significantly compromise the survival of the microencapsulated Gram-negative bacteria, contrary to the generally held view that spray-drying is generally too harsh of a process for preparing dry inoculant formulations for agriculture.^1,15^ Only a few other studies demonstrate successful use of spray-drying to encapsulate Gram-negative plant-beneficial bacteria. *Pseudomonas fluorescens-putida* microencapsulated in modified starch, ethyl cellulose, or methacrylic copolymer experienced a two-log reduction in population during spray-drying.^26^ *Enterobacter* microencapsulated in maltodextrin-alginate blends experienced post spray-drying survival between 50% and 80%, although the microcapsules contained minute amounts of alginate, and the polymer was not cross-linked.^27^

Drying processes (even those considered gentler than spray-drying) typically result in some reduction of microbial viability. For example, the drying step used in the conventional alginate encapsulation process has been reported to induce cell death. Lyophilization of alginate beads resulted in a 3 to 4 log reduction of encapsulated *Azospirillum brasilense*, and forced air drying at 30°C resulted in an even greater population decline.^12^ Bacterial survival depends heavily on the conditions experienced during the drying process.^40^ In the spray-drying procedure used in this study, bacteria were rapidly dehydrated as atomized droplets fell through the evaporation chamber. During drying, the surface temperatures of the droplets were limited to the wet bulb temperature, which was considerably less than the 130°C dry bulb temperature at the inlet. Once dry, the microcapsules accumulated in the collection chamber, where they were continually exposed to the 49–53°C outlet temperature. While these processing conditions did not significantly reduce the viability of microencapsulated *M. radiotolerans*, other microbes may be more susceptible. In order to mitigate population decline when encapsulating other microorganisms in CLAMs, the outlet temperature may be reduced by decreasing the inlet temperature or by increasing the product flow rate. Furthermore, in industrial-scale spray-dryers, the continual removal of powder at the outlet would limit exposure to elevated temperatures.

### Long-Term Viability of PPFMS in CLAMs

**Fig. 7.**
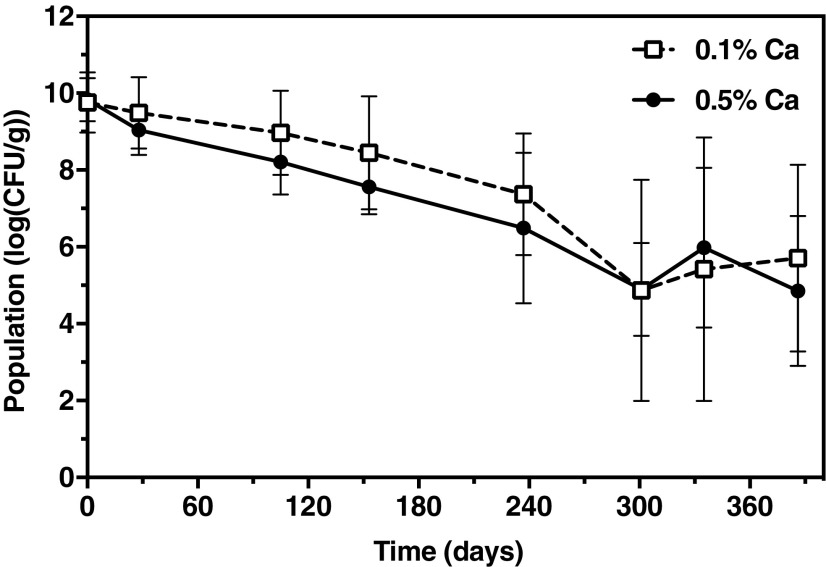
Survival of PPFM in CLAMs over one year of storage under ambient bench top conditions. Error bars indicate standard deviations from triplicate lots of 0.1% Ca and 0.5% Ca PPFM-CLAMs.

The population of PPFMs in CLAMs was monitored periodically over the course of one year of bench top storage. The average viable population declined gradually over the course of 12 months, with 0.1% Ca CLAMs experiencing a 4 log CFU/g reduction and 0.5% Ca CLAMs experiencing a 5 log CFU/g reduction (*Fig. 7*). The triplicate batches of both formulations experienced considerable variation in surviving PPFM population, particularly beyond 6 months of storage. In individual batches, PPFM populations exceeding 10^6^ and as high as 4.8 × 10^7^ CFU/g have persisted over one year of storage in CLAMs. However, replicate lots with populations below 10^4^ CFU/g decreased the average survival values and led to the high variances. The CaHPO_4_ content did not appear to influence the survival of PPFMs in CLAMs over the storage period.

In general, microbes in solution experience very limited shelf life, with the possible exception of those contained in water-in-oil emulsions.^41^ Microencapsulation in dry powder is a promising strategy to prolong bacterial viability during storage while reducing costs associated with transporting and storing large fluid volumes. Dry alginate has been reported to stabilize bacteria over the long term, with a considerable population remaining even after 14 years.^42^ The strategy employed in this study shows promise for achieving extended shelf-stability of PPFMs without specifically amending the CLAMs formulation. Future work in this area will be to explore formulation amendments to improve and extend bacterial stability in the dry microcapsules.

## Conclusion

This study demonstrates the microencapsulation of Gram-negative, plant-beneficial bacteria in cross-linked alginates by an industrially scalable single-step process that is immediately compatible with existing encapsulation technology. Spray-drying is a well-established, economical, and highly scalable encapsulation process. We demonstrated the successful microencapsulation of PPFMs in dry cross-linked alginate: both the viable bacteria loading and the bacterial survival upon spray-drying matched or exceeded the values previously reported with other organisms in dry alginate microbeads prepared by the conventional multistep gelation and drying method. There was no significant loss of PPFM viability upon spray-drying. The population of *M. radiotolerans* in PPFM-CLAMs declined gradually over one year of storage. The size of PPFM-CLAMs suggests compatibility with seed coating and foliar spray applications. Finally, tuning the extent of alginate cross-linking appears to offer a means of adjusting the release of bacterial cargo without influencing bacterial viability or particle size. Further research is warranted to investigate the application of PPFM-CLAMs to crops, particularly to assess the release of PPFMs in soil, their subsequent colonization of the plant, and their impact on crop yields.
